# Advertising Department

**Published:** 1873-07

**Authors:** 


### PART III.

### EDITORIAL AND MISCELLANEOUS.

*Advertising Department.*

As we have said again and again, a medical journal can never fully accomplish its great mission in meeting the wants and demands of the profession without an advertising department. Hence, we call the attention of our readers to ours. We solicit none but first class houses, and no others shall find admission into our columns knowingly. We have reason to believe that the RECORD has the largest circulation of any similar publication ever published in the South, and is second only to two or three in the United States. It is represented in its editorial staff by a number of the ablest medical men in almost all the Southern States and many of the Western and Northern, who feel and take a great interest in every department of the journal—indeed, with every thing calculated to further its interest or benefit its readers. Hence, it is the best medium for the Middle, Southern and Western States for advertising drugs, chemicals, books, philosophical apparatus, surgical instruments, etc. Our physicians and druggists require many things which advertisers can bring immediately and advantageously before them, through our journal, and it can be made a valuable agent and of the greatest mutual advantage both to the advertiser and those among whom it circulates. We ask none but first-class houses—these we can heartily and cordially indorse.

